# Study on the Frost Resistance of Concrete Modified with Steel Balls Containing Phase Change Material (PCM)

**DOI:** 10.3390/ma14164497

**Published:** 2021-08-11

**Authors:** Xiaosa Yuan, Baomin Wang, Peng Chen, Tao Luo

**Affiliations:** 1 Shaanxi Key Laboratory of Safety and Durability of Concrete Structures, Xijing University, Xi’an 710123, China; yuanxiaosa2009@163.com (X.Y.); chenpeng@xijing.edu.cn (P.C.); luotao19870426@126.com (T.L.); 2 School of Civil Engineering, Dalian University of Technology, Dalian 116023, China

**Keywords:** phase change concrete, macro-encapsulation, frost resistance, industrial CT, pore structure

## Abstract

In order to investigate the effect of phase change materials on the frost resistance of concrete in cold regions, hollow steel balls were used in this paper for the macroscopic encapsulation of the phase change material to replace some of the coarse aggregates in the preparation of phase change concrete. On the premise of ensuring reasonable mechanical properties, concrete mixed with different contents and different surface treatments of grouting steel balls were tested for the compressive strength and splitting tensile strength to determine the optimum content of phase change steel balls and investigate the frost resistance of phase change concrete. At the same time, industrial CT was used to explore the internal pore evolution pattern of concrete during the freeze–thaw period. The test results show that the optimum content of steel balls is 75%; during the freeze–thaw process, the mass loss, relative dynamic elastic modulus loss, and strength loss of phase change concrete are all lower than those of ordinary concrete, and the increase in porosity of phase change concrete is also significantly lower than that of ordinary concrete; the addition of phase change materials can optimise the distribution of the internal pore in concrete, improve its internal pore structure, and enhance its frost resistance.

## 1. Introduction

Phase change materials can make use of latent heat to store energy, and these materials can store or emit large amounts of heat through changes in their phase state to regulate the room temperature [1,2]. According to the form in which the phase change occurs, phase change materials can be divided into four categories: liquid–gas, solid–gas, solid–liquid, and solid–solid. Among them, solid–liquid phase change materials are easier to produce and use in large quantities due to their characteristics, including a large temperature range, a large amount of heat released during the phase change, the low production costs, and a variety of types for selection and use. However, if the solid–liquid phase change material is mixed directly with the building material without encapsulation, it is prone to leakage during the energy conversion process, which affects its use and durability [3].

The encapsulation of phase change materials is generally carried out by physical mixing methods, including macro-encapsulation and micro/nano-encapsulation, but the latter is too expensive and may have a negative impact on the mechanical properties of the corresponding structure, as well as building materials. In contrast, the macro-encapsulation of phase change materials in carriers such as light aggregates, spheres, and slabs, which are larger in volume, compared to micro/nano-encapsulated carriers, does not have much impact on the structural function of the building and has the advantages of simple production methods, low costs, the direct replacement of concrete aggregates, etc. As a result, this technology has received widespread interest from various studies [4,5,6,7,8,9,10,11].

In recent years, phase change materials have been used in buildings for energy efficiency, both for comfort and for reducing energy losses [12,13]. Hadjieva et al. [14] evaluated the thermal storage properties, structural stability, and the practicality of a PCM wallboard for heat storage made by applying thiosulphate pentahydrate to concrete. Koschenz et al. [15] designed an energy-efficient ceiling for office and industrial buildings, for which simulations and experimental validation were carried out. Karim et al. [16] used the thermal storage capacity of paraffin wax to make a lightweight insulated energy-saving flooring with light weight and heat preservation. Ryms et al. studied the application of PCM in modifying building materials by using different carriers [17,18]. Mazzucco et al. also studied the coupled behaviour of concrete modified by PCM particles employed as solid energy storage media [19].

In terms of temperature control in mass concrete, the incorporation of phase change materials can help to transfer the accumulated heat of hydration within mass concrete [20], alleviating and improving the cracking of concrete caused by large temperature differences. Kim et al. [21] mixed the barium salt phase change material into concrete to substantially reduce the heat of hydration of concrete. Qian et al. [22] studied the cooling effect of phase change materials instead of water as a coolant for concrete and pointed out that phase change materials have an important role in regulating the temperature rise and fall caused by cement hydration.

As icing on road surfaces has been difficult to tackle effectively in winter, especially in cold regions, many scientists have conducted in-depth research in recent years on using phase change materials to speed up ice and snow melting on pavements. Nayak et al. [23] used finite elements to evaluate the effect of phase change materials on the thermal effect of concrete pavements in winter, and the simulation results showed that the effect was better after adding phase change materials. Bentz et al. [24] earlier proposed that phase change materials can be mixed with cement-based materials to create a phase change functional layer in the pavement structure, so as to prevent or delay the formation of ice and frost by using the heat storage and exothermic properties of phase change materials. At present, there is more research on phase change materials in certain areas, including energy saving in buildings, temperature control in mass concrete, and snow melting in road surfaces, but less on improving the frost resistance of concrete; therefore, there is an urgent need for further work.

This research aims at studying the effect of PCM on the freeze–thaw resistance of concrete by using macroscopic encapsulation of PCM. Hollow steel balls containing phase change material were used to replace some of the coarse aggregates to modify concrete with guaranteed mechanical properties and the maximum amount of PCM added. On the premise of ensuring reasonable mechanical properties, concrete mixed with different contents and different surface treatments of grouting steel balls were tested for mechanical properties to determine the optimum admixture of phase change steel balls. Furthermore, rapid freeze–thaw tests were conducted on phase change concrete, and its freeze-resistance performance was studied by comparing and analysing the loss of mass, relative dynamic elastic modulus, and mechanical properties during freeze–thaw. In addition, industrial CT was used for nondestructive testing of concrete, and an in-depth analysis of the effect of phase change concrete on internal pore evolution and pore distribution during freeze–thaw was carried out.

## 2. Materials and Methods

### 2.1. Raw Materials

PO42.5 ordinary silicate cement was used, its chemical composition and related basic properties are shown in Table 1 and Table 2, respectively. The grade of fly ash used was class I, and its chemical composition and physical properties are shown in Table 1 and Table 3, respectively. The water reducing agent and air-entraining agent produced by Subote New Materials Co. Ltd. (Jiangsu, China) were used as admixtures. The water used for the test was ordinary tap water. The size of the gravel with continuous grading was 5~30 mm. The fine aggregate was river sand, with a fineness modulus of 2.58.

### 2.2. Encapsulation and Preparation of Phase Change Steel Balls and Grouting Steel Balls

The main component of the phase change material used in this test was tetradecane, which accounted for over 95%. The latent heat of phase change for the organic phase change material with a phase change temperature of 2 °C was found to be about 191.59 J/g. The hollow steel balls made of stainless steel 304 with an inner diameter of 23 mm and an outer diameter of 25 mm were used to encapsulate the phase change material. A 1 mm diameter hole was made in the upper part of the balls, and the balls were filled with phase change material through a syringe and then sealed by welding. Compared to other carrier materials such as ceramic pellets, the steel ball has the characteristics of a strong ability to contain phase change material and a good sealing effect; therefore, it is not easy to cause phase change material leakage. The steel ball was fully filled with PCM, the density of the phase change steel ball was 1743 kg/m^3^, and the mass of phase change material was 63% of the steel-ball-containing PCM.

For the preparation of grouting steel balls, the hollow steel balls with the same inner diameter and outer diameter were used. A hole with a diameter of 6 mm was made in the upper part of steel balls, and then the balls were filled with cement slurry. After the cement slurry had dried slightly, the total mass of the hollow steel ball and the cement slurry was weighed to obtain a density of 2439 kg/m^3^ for the grouting steel ball.

### 2.3. Preparation of Concrete Mixed with Phase Change Steel Balls

In this paper, 75% volume of large coarse aggregate (20~30 mm) was replaced by an equal volume of phase change steel balls to prepare phase change concrete, and a control group was set up as shown in Table 4.

### 2.4. Preparation of Concrete Mixed with Grouting Steel Balls

In this test, in order to investigate the effect of grouting steel balls with different surfaces on mechanical properties of concrete, the surface of a portion of the balls was treated with a metal repairing agent, thus classifying the grouting steel balls into two types: smooth surface and rough surface.

Grouting steel balls were used to replace 25%, 50%, 75%, and 100% volume of large coarse aggregate (20~30 mm) by equal volume, corresponding to the mix ratio design of concrete, as shown in Table 5.

All concrete specimens were demoulded at 24 h after casting and cured in standard curing boxes for 28 days.

### 2.5. Test Design

#### 2.5.1. Mechanical Performance Test

Compressive and splitting tensile tests were carried out on concrete with four different contents of grouting steel ball in the paper, and the test results were analysed to determine the optimum content for subsequent frost resistance tests on concrete admixed with phase change steel balls.

**Figure 1:**
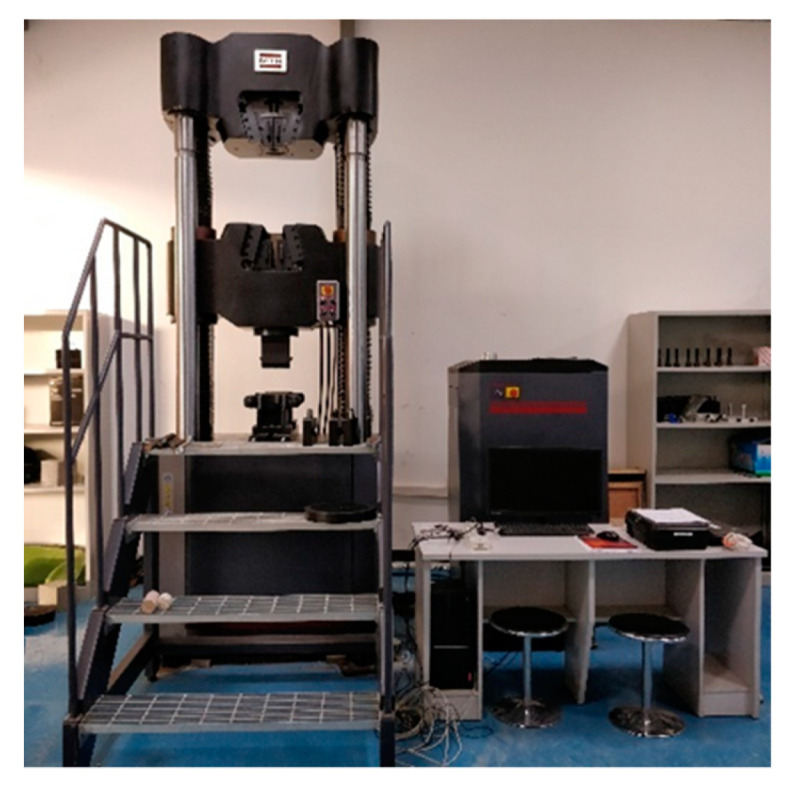
MTS universal testing machine.

The specimen size chosen for compressive and splitting tensile tests was 100 mm × 100 mm × 100mm, with reference to GB/T50081-2019. MTS universal testing machine (Shanghai, China) was used, shown in Figure 1. The loading speeds for compressive and splitting tensile tests were 0.5 MPa/s and 0.05 MPa/s, respectively.

#### 2.5.2. Rapid Freeze–Thaw Test

**Figure 2:**
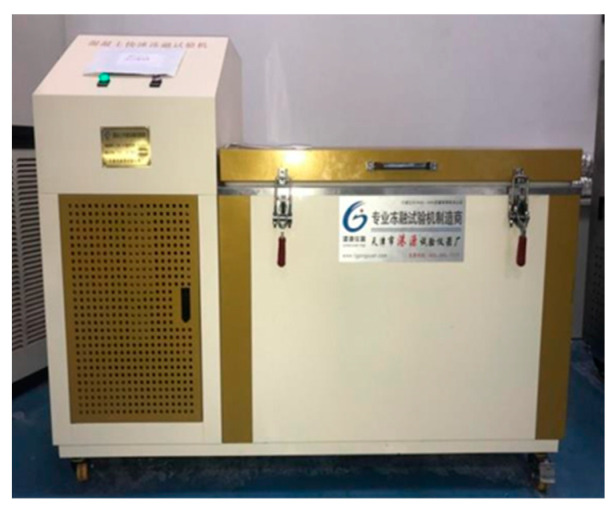
Rapid freeze–thaw test machine for concrete.

The concrete with the appropriate amount of phase change steel balls was prepared by referring to the results of the mechanical properties test. A rapid freeze–thaw test was carried out according to the test method specified in GBT50082; the equipment produced by Tianjin Gangyuan Test Instrument Co. Ltd. (Wenzhou, China) used is shown in Figure 2. Three specimens were used for each freeze–thaw cycle condition. Five freeze–thaw cycle conditions were considered, i.e., 0, 50, 100, 150, and 200 freeze–thaw cycles.

**Figure 3:**
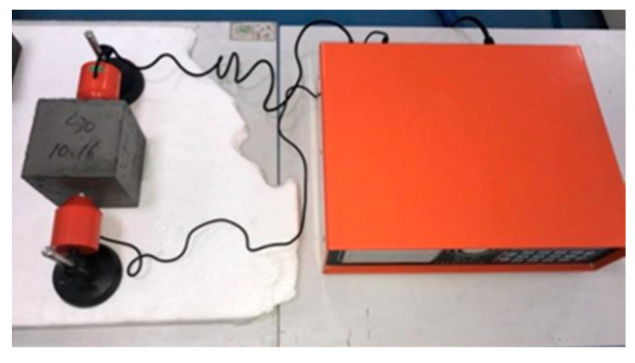
Measuring the dynamic modulus of elasticity.

The specimens with dimensions of 100 mm × 100 mm × 100 mm were immersed in water for 4 days and then removed for the freeze–thaw cycle test. During the test, the temperature in the centre of specimens was controlled from a minimum of (−18 ± 2) °C to a maximum of (5 ± 2) °C. Before the freeze–thaw test and after every 50 freeze–thaw cycles, the mass and the dynamic modulus of elasticity, as well as the strength, were measured. The mass loss, the relative dynamic modulus of elasticity, and the strength loss were calculated from the average of three specimens. The DT-20 dynamic elastic modulus tester produced by Tianjin Gangyuan Test Instrument Co. Ltd. (Wenzhou, China) was used, as shown in Figure 3.

#### 2.5.3. Industrial CT Inspection

Industrial CT (Multiscale-Voxel 450) produced by Sanying Precision Instruments Co., Ltd. (Tianjin, China) was employed to scan concrete specimens, and the CT data were processed using Avizo software to investigate the changes in the internal pore structure of phase change concrete and ordinary concrete under different freeze–thaw cycles. For both ordinary concrete and phase change concrete, only one specimen from each was scanned by CT with 0, 50, 100, 150, and 200 freeze–thaw cycles. In order to decrease the error caused by the boundary, the 100 mm × 100 mm × 100 mm specimen was cut into 80 mm × 80 mm× 80 mm in the postprocessing and pore structure analysis. After the samples were scanned by CT, Voxel Studio Recon software was used for reconstruction. The reconstruction resolution was 1100 × 1100 × 1100 PPI. Before the industrial CT scanning, the instrument self-examination and parameter setting work were carried out. After setting the voltage as 385 kV and current as 1.3 mA, the DR Film was taken for correction. The correction scanning was performed before every CT scanning, and parameters were set as the same for every scanning.

## 3. Results and Discussion

### 3.1. Compressive Strength of Concrete Mixed with Grouting Steel Balls

The variation in compressive strength of concrete mixed with different contents and different surfaces of grouting steel balls is shown in Figure 1 and Table 6 and Table 7, where P denotes ordinary concrete, G and C represent concrete with smooth and rough surface grouting steel balls, respectively, Y is the compressive test, and the figure in the specimen number indicates the volume fraction of large coarse aggregate replaced by grouting steel balls.

**Figure 4:**
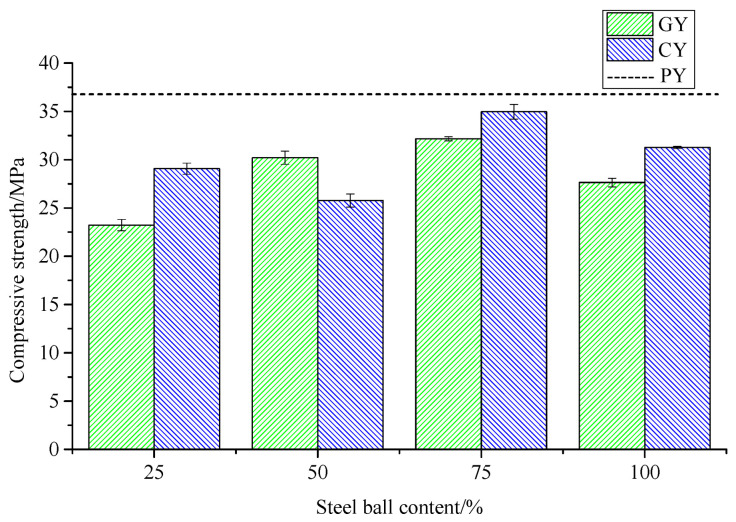
Change of compressive strength of grouted steel ball concrete.

It can be concluded from Table 6 and Table 7 and Figure 4 that the incorporation of steel balls could reduce the compressive strength of concrete to different degrees. The compressive strength of the concrete tends to increase and then decrease as the amount of grouting steel balls is increased. Compared to smooth steel balls, rough steel balls strengthen the bond between the ball surface and the cement slurry and their compressive strength is superior to that of concrete mixed with smooth steel balls in general. Compared to ordinary concrete, the compressive strength decreases the least when the steel balls are mixed at 75%, at which point the compressive strength of concrete mixed with smooth and rough steel balls decreases by 12.89% and 5.31%, respectively. The compressive strength results show that when using rough steel ball modified concrete with a volume replacement rate of 75%, the mechanical properties of the concrete are better and basically close to those of ordinary concrete.

### 3.2. Splitting Tensile Strength of Concrete Mixed with Grouting Steel Balls

**Figure 5:**
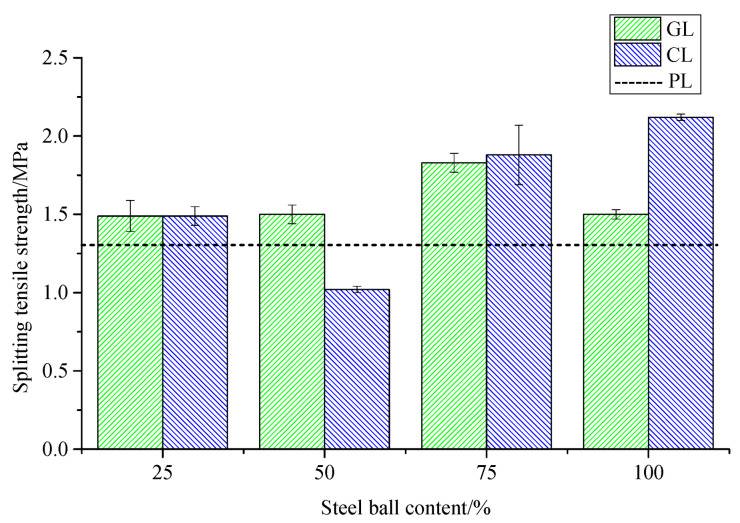
Change of splitting tensile strength of grouted steel ball concrete.

The effects of grouting steel balls with different dosing and surface treatments on splitting tensile strength of concrete are shown in Table 8 and Table 9 and Figure 5, where P denotes ordinary concrete, G and C represent the concrete mixed with smooth and rough grouting steel balls, respectively, L is splitting tensile test, and the figure in the specimen number indicates the volume fraction of large coarse aggregate replaced by grouting steel balls.

According to Table 8 and Table 9 and Figure 5, the incorporation of steel balls generally increases the splitting tensile strength of concrete. Similar to the compressive strength law, the splitting tensile strength of concrete mixed with rough steel balls is generally better than that with smooth steel balls. When the content of the smooth steel ball is 75%, the splitting tensile strength reaches the peak value of 1.83 MPa, with an increase of 39.69%; the splitting tensile strength with 75% rough steel balls is 1.88 MPa, with an increase of 43.51%. Therefore, the splitting tensile strength of the two kinds of concrete is almost the same.

Based on the above compressive strength and splitting tensile strength results, the optimum content of grouting steel balls is initially determined to be 75%, and the rough surface of the steel ball is more effective in modifying the concrete; the conclusions were applied to the subsequent freeze–thaw test.

### 3.3. Frost Resistance of Concrete Mixed with Phase Change Steel Balls

#### 3.3.1. Surface Spalling

**Figure 6:**
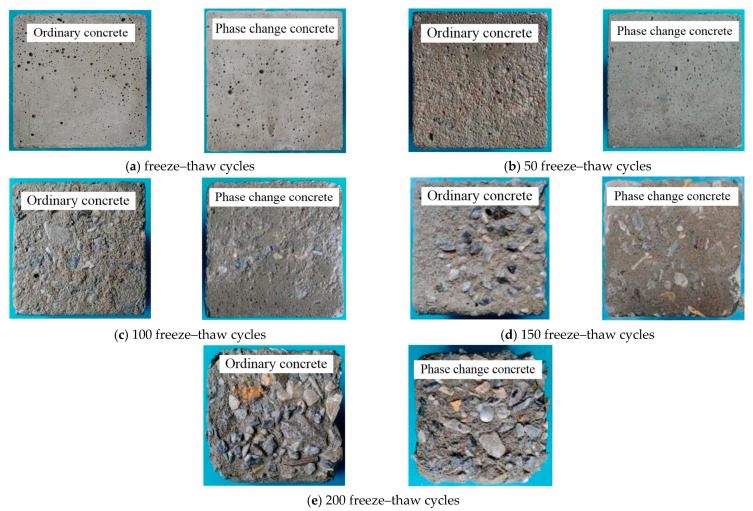
Comparison of concrete surface spalling after freezing and thawing.

Figure 6 shows the surface spalling of phase change concrete and ordinary concrete after 50, 100, 150, and 200 freeze–thaw cycles, respectively, demonstrating that the surface spalling of phase change concrete is better than that of ordinary concrete after the same number of freeze–thaw cycles.

#### 3.3.2. Mass Loss and Relative Dynamic Modulus of Elasticity

**Figure 7:**
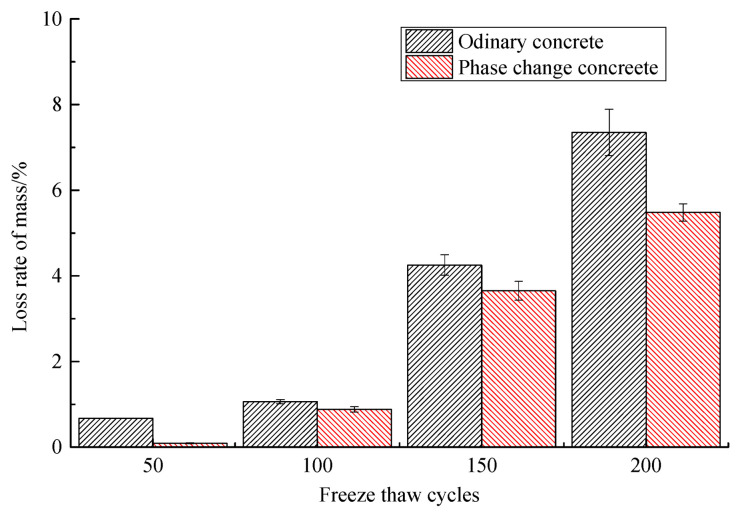
Change of mass loss rate of concrete during freeze–thaw cycles.

**Figure 8:**
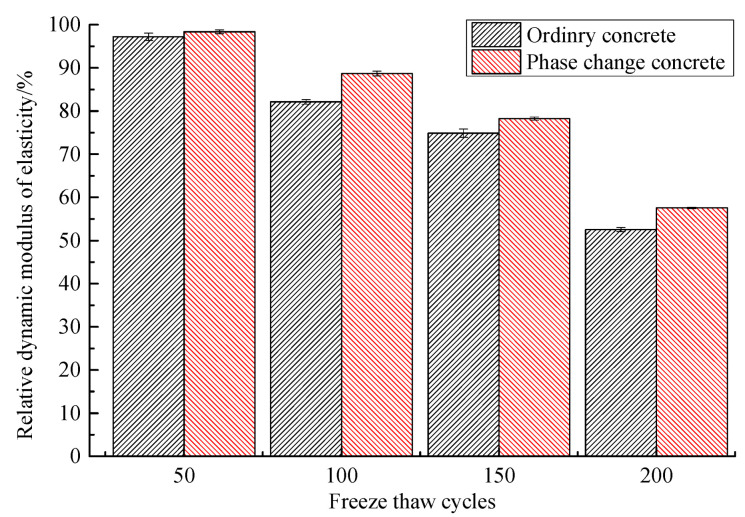
Comparison of relative dynamic elastic modulus.

Based on the comparison of the mass loss rate and relative dynamic elastic modulus of ordinary concrete and phase change concrete during freeze–thaw, presented in Table 10 and Table 11 and Figure 7 and Figure 8, it can be concluded that the phase change material can reduce the mass loss and relative dynamic elastic modulus loss of concrete to a certain extent. At the same number of freeze–thaw cycles, the mass loss rate and relative dynamic elastic modulus loss of phase change concrete are lower than those of ordinary concrete, and its frost resistance is better. After 50 freeze–thaw cycles, the mass loss rate of ordinary concrete is much higher than that of phase change concrete, indicating that the phase change material is effective in improving the frost resistance of concrete in the early stage. In terms of the limit of concrete to withstand freeze–thaw cycles, the ordinary concrete will soon enter failure after 150 freeze–thaw cycles, while the phase change concrete does not occur until the freeze–thaw cycle is close to 200 times.

#### 3.3.3. Mechanical Properties

**Figure 9:**
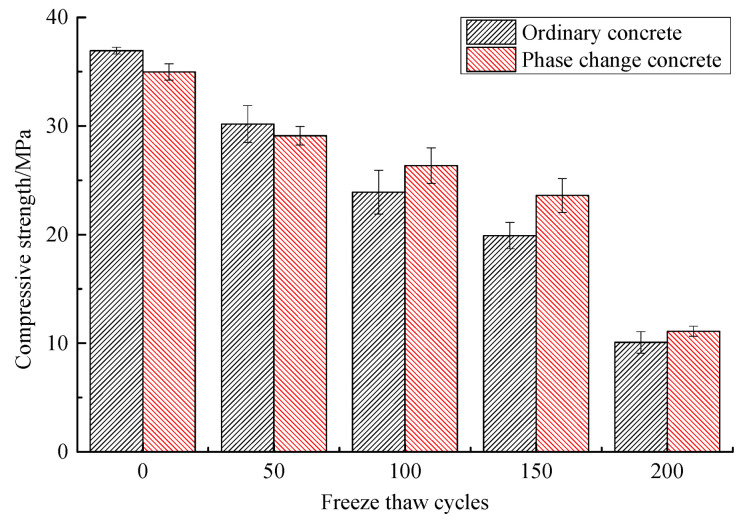
Change of compressive strength during freeze–thaw period.

**Figure 10:**
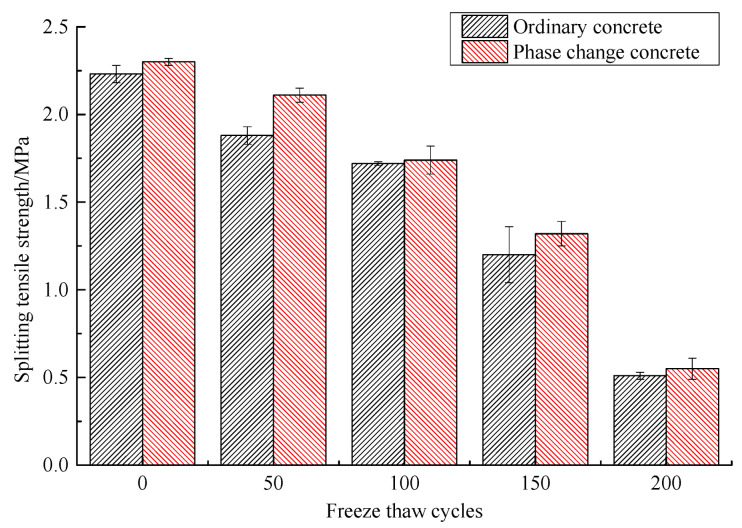
Variation of splitting tensile strength with freeze–thaw cycles.

The changes in compressive strength of concrete during freeze–thaw are shown in Table 12 and Figure 9, and the changes in the splitting tensile strength are shown in Table 13 and Figure 10. As can be seen from these graphs, the compressive and the splitting tensile strength of concrete continue to decrease as freeze–thaw cycles increase, but the addition of phase change material reduces the loss of mechanical properties to some extent. After 50 freeze–thaw cycles, the compressive strength loss of phase change concrete and ordinary concrete is 16.79% and 18.30%, respectively, which is not a big difference, while the splitting tensile strength of the former improves more obviously at this time. After 100 freeze–thaw cycles and 150 freeze–thaw cycles, the compressive strength loss rate of phase change concrete is 24.65% and 32.51%, respectively, while that of ordinary concrete reaches 35.28% and 46.11%, and the difference between the two increases significantly. After 200 freeze–thaw cycles, the difference in the compressive strength and splitting tensile strength loss rate between the two is not significant, but the compressive strength of phase change concrete is still greater than that of ordinary concrete.

### 3.4. Analysis of the Pore Structure in Concrete

#### 3.4.1. Porosity

Table 14 shows the changes in porosity (percentage of pore volume) of ordinary concrete and phase change concrete during the freeze–thaw process, from which it can be seen that the total porosity of these two kinds of concrete gradually increases as the freeze–thaw cycle continues. The porosity growth rate of phase change concrete at 150 freeze–thaw cycles is still significantly lower than that of ordinary concrete at 100 freeze–thaw cycles, and the difference in porosity growth rate between the two types of concrete also reaches its maximum at 150 freeze–thaw cycles, approximately 24%, at which point the improvement in the internal deterioration of phase change concrete is the most obvious. Throughout the whole freeze–thaw process, the change in porosity of phase change concrete is significantly less than that of ordinary concrete, indicating that phase change concrete is less deteriorated and that the addition of phase change materials can result in a significant improvement in frost resistance of concrete.

#### 3.4.2. Pore Distribution

**Figure 11:**
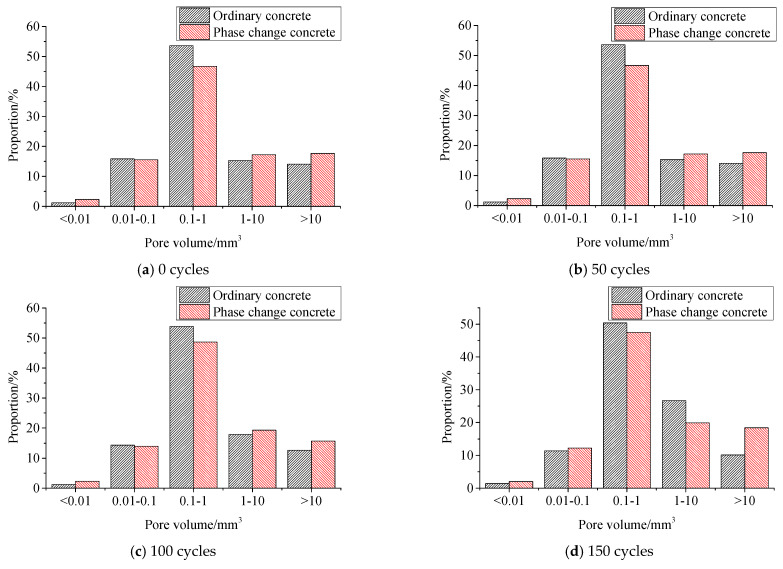

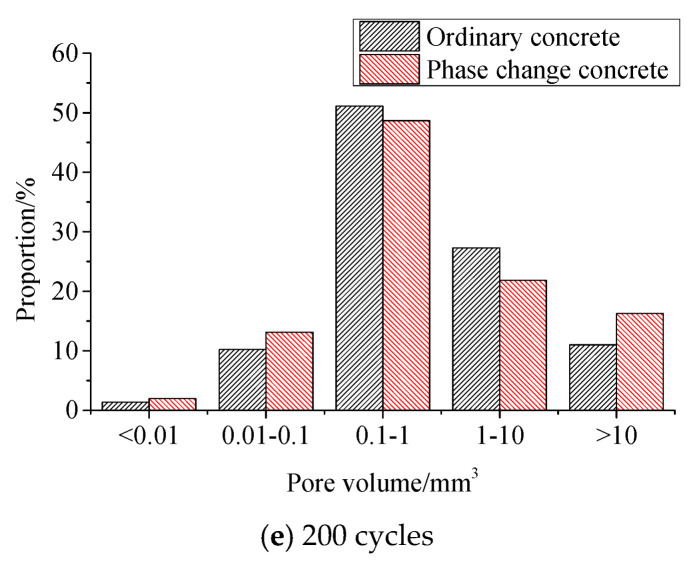
Comparison of pore volume ratio between ordinary concrete and phase change concrete under different freeze–thaw cycles.

Table 15 and Figure 11 show the volume proportion of pores in different size ranges during freeze–thaw.

It can be seen from Table 15 that in ordinary concrete and phase change concrete, the pore volume ratio of 0.1~1 mm^3^ accounts for the most, while that of less than 0.01 mm^3^ accounts for the least, and it is similar in the other three ranges.

As freeze–thaw cycles increase, the proportion of pores within 0.01~0.1 mm^3^ in ordinary concrete decreases, and the proportion of pores within 1~10 mm^3^ increases continuously; the proportion of pores with the volume of 0.1~1 mm^3^ fluctuates; the proportion of pores with a volume greater than 10 mm^3^ gradually decreases before 150 freeze–thaw cycles and rebounds slightly at 200 cycles, with the pattern of change being the opposite of that of less than 0.01 mm^3^ pores. In the same case, the proportion of pores with the volume of 0.1~1 mm^3^ in phase change concrete tends to increase and then decrease, and fluctuations occur in all four other ranges.

From Figure 11, the pore distribution difference of the five size ranges in ordinary concrete during freeze–thaw is greater than that of phase change concrete, and therefore, it can be concluded that the addition of phase change materials can improve the internal pore structure of concrete, making the internal pore distribution more uniform.

## 4. Conclusions

In this paper, the mechanical properties of concrete mixed with grouting steel balls were studied. The optimum content of steel balls was determined under the premise of ensuring the reasonable mechanical properties of concrete. Additionally, phase change materials were prepared into phase change coarse aggregate by being packed into hollow steel balls and then encapsulated macroscopically, so as to prepare phase change concrete according to the optimal dosage of steel ball in the frost resistance effect research. The main conclusions are as follows:

(1) The incorporation of grouting steel balls can reduce compressive strength and increase the splitting tensile strength of concrete to varying degrees, and the balls with the rough surface have a better effect on the modification of concrete; combining the results of compressive strength and splitting tensile strength, the optimum dosing of grouting steel balls can be initially determined to be 75%.
(2) During the freeze–thaw process, all the freeze-resistance indexes of phase change concrete are better than those of ordinary concrete. In the early freeze–thaw period (50 cycles), the difference in compressive strength loss between ordinary concrete and phase change concrete is not significant, while the improvement in splitting tensile strength of phase change concrete is more obvious at this time. In the middle freeze–thaw period (100–150 freeze–thaw cycles), the difference between them increases greatly and the advantage of phase change materials in improving the reduction of compressive strength is significant. In the late freeze–thaw period (200 freeze–thaw cycles), the difference in strength loss between the two types of concrete is not obvious.
(3) Throughout the whole freeze–thaw process, the change in porosity of phase change concrete is significantly lower than that of ordinary concrete, and its internal deterioration is lesser. The addition of phase change materials optimises the pore structure distribution in concrete and improves the internal pore structure.

## Figures and Tables

**Table 1:** Chemical composition of cement (%).

Type	CaO	SiO_2_	Al_2_O_3_	Fe_2_O_3_	TiO_2_	MgO	SO_3_	Na_2_O	Ignition Loss
Cement	8.93	48.50	25.36	5.12	0.57	1.15	1.15	0.52	0.79
Fly ash	4.88	49.02	31.56	6.97	-	0.83	1.2	-	3.65

**Table 2:** Basic properties of cement.

Normal Consistency (%)	Setting Time (h)		Stability	Compressive Strength (MPa)		Flexural Strength (MPa)		Density (g/cm^3^)
	Initial	Final		3 Days	28 Days	3 Days	28 Days	
25.3	1:38	2:33	Qualified	18.3	39.3	2.6	6.2	3.11

**Table 3:** Physical properties of fly ash.

Specific Surface Area (m^2^/kg)	Apparent Density (g/cm^3^)	Fineness (45 μm)	Water Content	Water Demand Ratio
0.994	2.56	18.0%	0.4%	88.0%

**Table 4:** Mix proportion of concrete modified by steel balls containing phase change material (kg/m^3^).

Type	C	FA	S	W	SBPCM	Coarse Aggregate			WRA	AEA
						Small	Medium	Large		
Ordinary concrete	202	87	688	130	0	389	505	355	1.1918	0.0202
Phase change concrete	202	87	688	130	214.5	389	505	88.75	1.1918	0.0202

C, cement; FA, fly ash; S, sand; W, water; SBPCM, steel-ball-containing PCM; WRA, water reducing agent; AEA, air-entraining agent.

**Table 5:** Mix proportion of concrete modified by grouting steel balls (kg/m^3^).

Type	C	FA	S	W	GSB	CA			WRA	AEA
						Small	Medium	Large		
Ordinary Concrete	202	87	688	130	0	389	505	355	1.1918	0.0202
Grouted steel ball concrete	202	87	688	130	100	389	505	266.25	1.1918	0.0202
	202	87	688	130	200	389	505	177.5	1.1918	0.0202
	202	87	688	130	300	389	505	88.75	1.1918	0.0202
	202	87	688	130	400	389	505	0	1.1918	0.0202

GSB, grouting steel ball.

**Table 6:** Compressive strength of grouted steel ball concrete.

No.	Compressive Strength (MPa)	No.	Compressive Strength (MPa)
PY	36.93 ± 0.31		
GY25	23.23 ± 0.59	CY25	29.07 ± 0.58
GY50	30.20 ± 0.69	CY50	25.77 ± 0.68
GY75	32.17 ± 0.21	CY75	34.97 ± 0.75
GY100	27.63 ± 0.45	CY100	31.27 ± 0.12

**Table 7:** Compressive strength loss rate of grouted steel ball concrete with different contents.

No.	Loss Rate (%)		
PY	0		
GY25	37.10	CY25	21.28
GY50	18.22	CY50	30.22
GY75	12.89	CY75	5.31
GY100	25.18	CY100	15.33

**Table 8:** Splitting tensile strength of grouted steel ball concrete.

No.	Splitting Tensile Strength (MPa)	No.	Splitting Tensile Strength (MPa)
PL	1.31 ± 0.05		
GL25	1.49 ± 0.10	CL25	1.49 ± 0.06
GL50	1.50 ± 0.06	CL50	1.02 ± 0.02
GL75	1.83 ± 0.06	CL75	1.88 ± 0.19
GL100	1.58 ± 0.03	CL100	2.12 ± 0.02

**Table 9:** Improvement of splitting tensile strength of grouted steel ball concrete.

No.	Improvement (%)		
PL	0		
GL25	13.74	CL25	13.74
GL50	14.50	CL50	−22.14
GL75	39.69	CL75	43.51
GL100	20.61	CL100	61.83

**Table 10:** Mass loss rate of concrete during freeze–thaw cycles (%).

Freeze–Thaw Cycles	Ordinary Concrete	Phase Change Concrete
50	0.67 ± 0	0.09 ± 0.01
100	1.06 ± 0.047	0.88 ± 0.06
150	4.25 ± 0.24	3.65 ± 0.22
200	7.35 ± 0.54	5.48 ± 0.20

**Table 11:** Relative dynamic elastic modulus of concrete (%).

Freeze–Thaw Cycles	Ordinary Concrete	Phase Change Concrete
0	100	100
50	97.21 ± 0.85	98.38 ± 0.46
100	82.12 ± 0.54	88.69 ± 0.56
150	74.90 ± 0.96	78.24 ± 0.37
200	52.53 ± 0.49	57.55 ± 0.16

**Table 12:** Compressive strength of concrete during freezing and thawing (MPa).

Freeze–Thaw Cycles	Ordinary Concrete	Phase Change Concrete
0	36.93 ± 0.31	34.97 ± 0.75
50	30.17 ± 1.70	29.10 ± 0.85
100	23.90 ± 2.01	26.35 ± 1.63
150	19.9 ± 1.21	23.60 ± 1.56
200	10.07 ± 1.00	11.10 ± 0.46

**Table 13:** Splitting tensile strength of concrete during freeze–thaw (MPa).

Freeze–Thaw Cycles	Ordinary Concrete	Phase Change Concrete
0	2.23 ± 0.05	2.30 ± 0.02
50	1.88 ± 0.05	2.11 ± 0.04
100	1.72 ± 0.01	1.74 ± 0.08
150	1.20 ± 0.16	1.32 ± 0.07
200	0.51 ± 0.02	0.55 ± 0.06

**Table 14:** Change of porosity in concrete during freeze–thaw process.

		0 Cycles	50 Cycles	100 Cycles	150 Cycles	200 Cycles
Ordinary concrete	Porosity (%)	2.41	2.91	3.47	3.97	4.61
	Increment (%)	-	20.75	43.98	64.73	91.29
Phase change concrete	Porosity (%)	1.64	1.90	2.14	2.44	2.87
	Increment (%)	-	15.85	60.49	40.78	75.00

**Table 15:** Volume proportion of pores in different size ranges in concrete (%).

Type	Cycles	Pores of Different Sizes				
		<0.01 mm^3^	0.01~0.1 mm^3^	0.1~1 mm^3^	1~10 mm^3^	>10 mm^3^
Ordinary concrete	0	1.20	15.84	53.59	15.29	14.07
	50	1.24	14.34	53.85	17.93	12.64
	100	1.38	12.57	51.17	24.09	10.78
	150	1.41	11.39	50.38	26.66	10.15
	200	1.35	10.24	51.11	27.28	11.02
Phase change concrete	0	2.29	15.53	46.69	17.21	17.65
	50	2.07	12.88	49.03	20.57	15.45
	100	2.30	13.99	48.66	19.35	15.70
	150	2.00	12.25	47.50	19.87	18.37
	200	1.98	13.12	46.80	21.85	16.25

## Data Availability

The data presented in this study are available on request from the corresponding author.
